# Power-law distribution in the number of confirmed COVID-19 cases

**DOI:** 10.1063/5.0013031

**Published:** 2020-09-14

**Authors:** Bernd Blasius

**Affiliations:** Institute for Chemistry and Biology of the Marine Environment, University of Oldenburg, 26111 Oldenburg, Germany

## Abstract

COVID-19 is an emerging respiratory infectious disease caused by the coronavirus SARS-CoV-2. It was first reported on in early December 2019 in Wuhan, China and within three months spread as a pandemic around the whole globe. Here, we study macro-epidemiological patterns along the time course of the COVID-19 pandemic. We compute the distribution of confirmed COVID-19 cases and deaths for countries worldwide and for counties in the US and show that both distributions follow a truncated power-law over five orders of magnitude. We are able to explain the origin of this scaling behavior as a dual-scale process: the large-scale spread of the virus between countries and the small-scale accumulation of case numbers within each country. Assuming exponential growth on both scales, the critical exponent of the power-law is determined by the ratio of large-scale to small-scale growth rates. We confirm this theory in numerical simulations in a simple meta-population model, describing the epidemic spread in a network of interconnected countries. Our theory gives a mechanistic explanation why most COVID-19 cases occurred within a few epicenters, at least in the initial phase of the outbreak. By combining real world data, modeling, and numerical simulations, we make the case that the distribution of epidemic prevalence might follow universal rules.

In this study, we address one of the striking characteristics of the COVID-19 pandemic, namely, the huge variation in the number of cases that have been reported from different parts of the world. In the first month of the outbreak, some countries, the so-called “epi-centers” of the pandemics, were already severely struck by the pandemic, whereas many others at the same time had just confirmed the first few cases. Similar heterogeneous distributions occurred on a smaller scale within countries and led to the paradoxical situation that despite the huge number of infected persons worldwide, many people (still) experienced a mild number of cases in their local neighborhood. To quantify this pattern, we analyze empirical data on the number of confirmed COVID-19 cases and show that the epidemic prevalence is distributed as a truncated power-law over many orders of magnitude. This indicates that the transition between the few epidemic epi-centers and the large number of weakly affected regions is scale-free and, thus, the strong inequality of reported cases is an expression of the fact that COVID-19 is geographically distributed as a fractal. Even though there are many factors that potentially are contributing to this spatial heterogeneity (e.g., idiosyncratic differences in sizes, geography, mitigation measures, and testing regimes in different countries), we develop a simple dynamic theory that is able to explain the reported data and indicates that the emergence of a power-law distribution is a natural outcome of the spreading process itself. Our theory also gives cause for concern: as the fractal distribution arises only in the initial phase of the pandemic, epidemic prevalence patterns might be very different if the pandemic breaks out again in a second wave after a prolonged lock-down period.

## I. INTRODUCTION

COVID-19 is an emerging infectious disease caused by the coronavirus SARS-CoV-2. It was first reported on in Hubei, mainland China on December 31, 2019 and has spread well outside China in a matter of a few weeks, reaching countries in all parts of the globe within a time span of three month. As of 29 March 2020, the disease has arrived in 177 countries, with more than 700 000 confirmed cases and 30 000 deaths worldwide.^42^ Despite the drastic, large-scale containment measures implemented in most countries, these numbers are rapidly growing every day—posing an unprecedented threat to the global health and economy of interconnected human societies.

One of the most powerful tools to understand the laws of epidemic growth is mathematical modeling, going back to Bernoulli’s work^5^ on the spread of smallpox in 1760. Epidemiological models can be roughly divided into two classes. The first class of models is focused on describing the temporal development of the epidemic within a localized region or country. These models are often variants of the well-known susceptible-infected-recovered (SIR) model^22,23^ and have recently been adapted to the situation of COVID-19, taking into account non-pharmaceutical interventions (e.g., quarantine, hospitalization, and containment policies) and allowing first predictions of healthcare demand.^15,25,26,40^

The second class of models is concerned with the geographic spread of the epidemic around the globe. For these aims, spatially explicit models have been developed that leverage information on the topology of transport networks. For example, the global network of cargo ship movements^21^ was used to model the dispersal of invasive species.^37^ Similarly, for infectious diseases, in a pioneering study, the 2003 spread of SARS in the global aviation network^41^ was modeled.^19^ Based on these approaches, conceptual frameworks have been developed to estimate epidemic arrival times as effective distances.^8,20^ At the same time, these models have been refined to highly detailed simulation frameworks for predicting the spread of disease and are able to include factors such as vaccination, multiple susceptibility classes, seasonal forcing, and the stochastic movement of individual agents.^12,43^ Reacting rapidly to the emergent pandemic, spatial epidemiological models have been developed to describe and anticipate the spread of COVID-19.^2,10,16,33^ These models allow us to predict the incidence of the epidemics in a spatial population through time, permitting to study the impact of travel restrictions and other control measures.

Despite this theoretical progress, not much is known about the biogeography of COVID-19, neither from empirical studies nor from mathematical models. This is astonishing, as one prominent characteristic of the pandemic is the huge variation in the number of cases that have been reported from different countries of the world. As of April 2020, some countries—the epicenters of the pandemic—were already badly affected by the pandemic, while others at the same time had just confirmed the first few cases. This geographic variation in COVID-19 prevalence might be explained by several arguments: a first obvious possibility would be that the variation is caused by the idiosyncratic circumstances of the individual countries which differ largely in their geography and population size, but also in the way they are combatting the disease. Alternatively, parts of the variation could simply be due to reporting errors, reflecting disparate national testing regimes, with countries such as China, Japan, South Korea, or Germany having high testing rates, in contrast to other countries with much poorer testing. Here, we argue, however, that a dominant part of this variation may be a direct consequence of the dynamics of the spreading process itself. Thereby, the epidemic prevalence in a country should be directly correlated to the arrival time of the disease: countries that were invaded very early by the virus have accumulated many cases in time, while countries with a late invasion naturally still have smaller prevalence.

To test this hypothesis, we use empirical data^14^ to compute the country-level distribution, P, of confirmed COVID-19 cases, n, at the end of March 2020 worldwide and find that it is closely approximated by a truncated power-law,

$$P(n)∼n^{-\mu },\;1\leq n\leq n_{max}$$ (1)

over five orders of magnitude.

Power-law distributions characterize a large range of phenomena in natural, economic, and social systems, which is known as Zipf- or Pareto law.^11,27,29,39^ Examples range from the number of species in biological taxa,^46^ the number of cities with a given size,^47^ the number of different words in human language,^47^ the frequency of earthquakes,^18^ the distribution of wealth,^32^ the number of scientific citations,^34,36^ the step length in animal search patterns,^44^ and the popularity of chess openings.^7^ Our study shows that epidemic prevalence, at least in the emerging stage of a pandemic, is another system that falls into this class, suggesting that the spatial distribution of COVID-19 case numbers is a fractal.^9^

The appearance of a power-law distribution often points to the nature of the underlying processes. It might, for example, be an indication that the system operates close to criticality,^3,29^ and it might hint at the presence of a multiplicative stochastic process with certain boundaries^7,39^ or a rich-get-richer process.^4,38^

Here, we provide a conceptual dual-scale model that explains the emergence of the power-law distribution by the “superposition” of two concurrent processes: large-scale spread of the virus between countries and small-scale snowballing of case numbers within each country. Assuming exponential growth on both scales, the critical exponent is simply determined by the ratio of large-scale to small-scale growth rates. We confirm this theory in numerical simulations in a simple meta-population model, describing the epidemic spread in a network of interconnected countries. By combining real world data, modeling, and numerical simulations, we make the case that the distribution of epidemic prevalence, and possibly that of spreading processes in general, might follow universal rules.

## II. RESULTS

### A. Power-law distribution in empirical data

Our research builds on the COVID-19 data repository operated by the Johns Hopkins University Center for Systems Science and Engineering (JHU CSSE).^14^ The database contains information about the daily number of confirmed COVID-19 cases and confirmed deaths in various countries worldwide.

Using these data, we computed the distribution $P_{C}(n)$ of confirmed cases and the distribution $P_{D}(n)$ of confirmed deaths at a given date (see Appendix A).

**FIG. 1.**
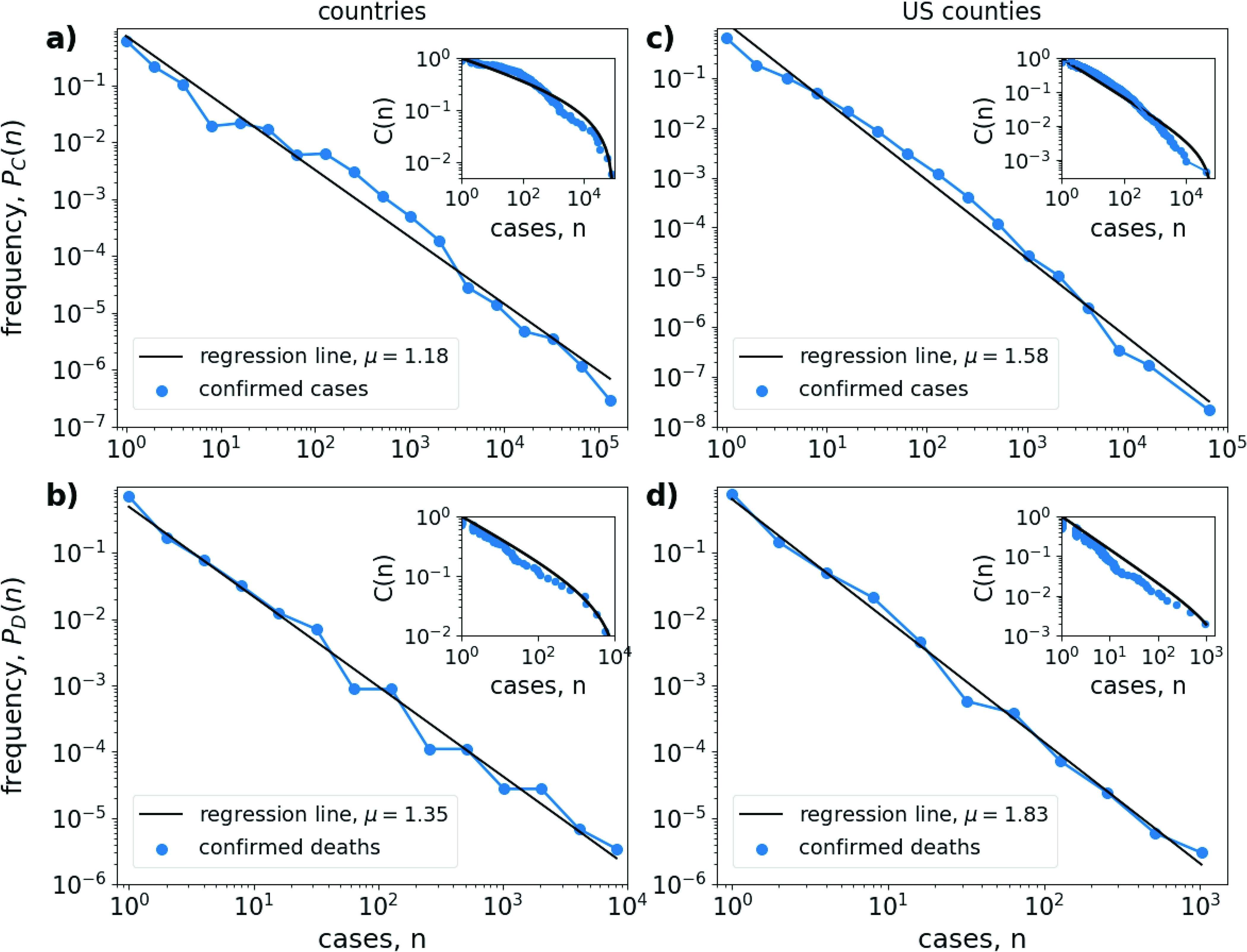
Power-law scaling in the distribution of confirmed COVID-19 cases. Left column: Estimated probability $P_{x}(n)$ (blue lines and circles) for a country to have a certain number n of (a) confirmed cases (x=C) and (b) confirmed deaths (x=D) on March 22, 2020. Right column: The same for the 2160 US counties that have been invaded by the coronavirus on March 31, 2020. Histogram bins are spaced equally on a logarithmic axis and only bins with a positive number of entries are shown. Black solid lines show straight-line fits with slope μ, indicated in the figure labels. Insets: Cumulative fraction $C(n)=\sum_{m=n+1}^{N}P(m)$ of countries, or counties, with case number m>n. Solid lines show the cumulative distribution equation (A2) of a truncated power-law distribution with critical exponent μ and cut-off value (a) $n_{max}=1\times 10^{5}$, (b) $n_{max}=1.5\times 10^{4}$, (c) $n_{max}=7\times 10^{4}$, and (d) $n_{max}=3\times 10^{3}$.

The country-level prevalence distribution on March 22, 2020 is shown in Figs. 1(a) and 1(b). On that day, 168 countries were invaded by the coronavirus and 86 countries already had reported fatalities. The number of confirmed cases varied between 81 435 cases in China (followed by 59 138 cases in Italy) and 1 case in 16 countries. The number of confirmed deaths varied between 5476 in Italy (followed by 3274 in China) and one or zero deaths in many countries.

Figures 1(a) and 1(b) clearly demonstrate that the frequency P of countries that have a certain number n of COVID-19 cases follows a broad, long-tailed distribution that in very good approximation can be described by a power-law, spanning five orders of magnitude for the confirmed number of cases and four orders of magnitude for the confirmed number of deaths.

To illustrate the robustness of our hypothesis to spatial scale, in Figs. 1(c) and 1(d), we depict the same analysis for the distribution of confirmed COVID-19 cases in US counties on March 31, 2020. On this day, 2160 counties were invaded by the virus and 514 counties reported at least on death. Epidemic prevalence varied between 43 119 confirmed cases and 922 confirmed deaths in New York City and one confirmed case in 455 counties and one confirmed death in 253 counties. Again, we find that the distribution of confirmed cases follows a power-law over several orders of magnitude. Thus, although the two datasets differ greatly in spatial scale and resolution [168 invaded countries in Figs. 1(a) and 1(b) vs 2160 invaded US counties in Figs. 1(c) and 1(d)], we obtain very similar patterns of prevalence distribution.

A crude estimation of the critical exponent can be obtained by measuring the slope of a regression line through the data on a double-logarithmic plot. Applying this method to the country-level distribution [Figs. 1(a) and 1(b)], we obtain a value of $\mu_{C}=1.18$ (slope of the distribution of confirmed cases) and $\mu_{D}=1.35$ (confirmed deaths). For the US-county distribution [Figs. 1(c) and 1(d)], we obtain somewhat larger slopes of $\mu_{C}=1.58$ and $\mu_{D}=1.83$.

A more accurate estimation of the critical exponent is provided by a maximum likelihood estimation (see Appendix A). Applying this approach to the country-level COVID-19 distribution yields critical exponents of ${\hat{\mu }}_{C}=1.14\pm 0.01$ and ${\hat{\mu }}_{D}=1.50\pm 0.05$. For the US-county distribution, we obtain the value ${\hat{\mu }}_{C}=1.49\pm 0.01$ and ${\hat{\mu }}_{D}=2.31\pm 0.06$. These exponents slightly deviate from those obtained from the regression analysis, but are still in the same ballpark.

Given an unbounded power-law distribution P(n), the cumulative distribution function $C(n)=\int_{n}^{\infty }P(n^{\prime })dn^{\prime }$ should also follow a power-law $C(n)∼n^{1-\mu }$. As shown in the insets in Fig. 1, this is not the case for the distribution of COVID-19 cases, for which the cumulative fraction $C(n)=\sum_{m=n+1}^{N}P(m)$ of countries, or counties, with case number m>n do not really follow a straight line in a double logarithmic plot. Instead, they are better described by the cumulative distribution function Eq. (A2) of a truncated power-law, that is, a power-law distribution with an upper bound $n_{max}$ for the number of cases, Eq. (1) (see Appendix A and Fig. 5). This indication for the presence of a truncated power-law distribution also conforms with our theoretical analysis below.

However, we note that although the shape of the empirically obtained C(n) overall follows the curve of a truncated power-law distribution, there is a considerable wavering around the theoretical curve (compare blue circles and black lines in Fig. 1 insets). Thus, a rigorous hypothesis testing with Monte Carlo simulations,^11,13^ which does not take disturbances due to additional irregularities (e.g., heterogeneities in country sizes or containment measures) fully into account, will always reject the hypothesis of a perfect truncated power-law as the true underlying distribution.

The presence of a power-law distribution means that global COVID-19 prevalence patterns are characterized by a small number of countries with huge epidemic prevalence (the long tail of the distribution) and a large number of countries that are (yet) barely affected by the disease. In between these two extremes, there is a smooth transition and this transition is scale-free, that is, the amplification in the number of countries (or counties) with decreasing number of cases is the same at all scales. In general, the obtained critical exponents are rather small. While for most natural power-law distributions critical exponents are around μ≈2, here we estimate exponents that are clearly below two, μ<2, indicating a very broad distribution for which in the absence of an upper bound, the mean value diverges.

### B. Temporal development during the pandemic spread

**FIG. 2.**
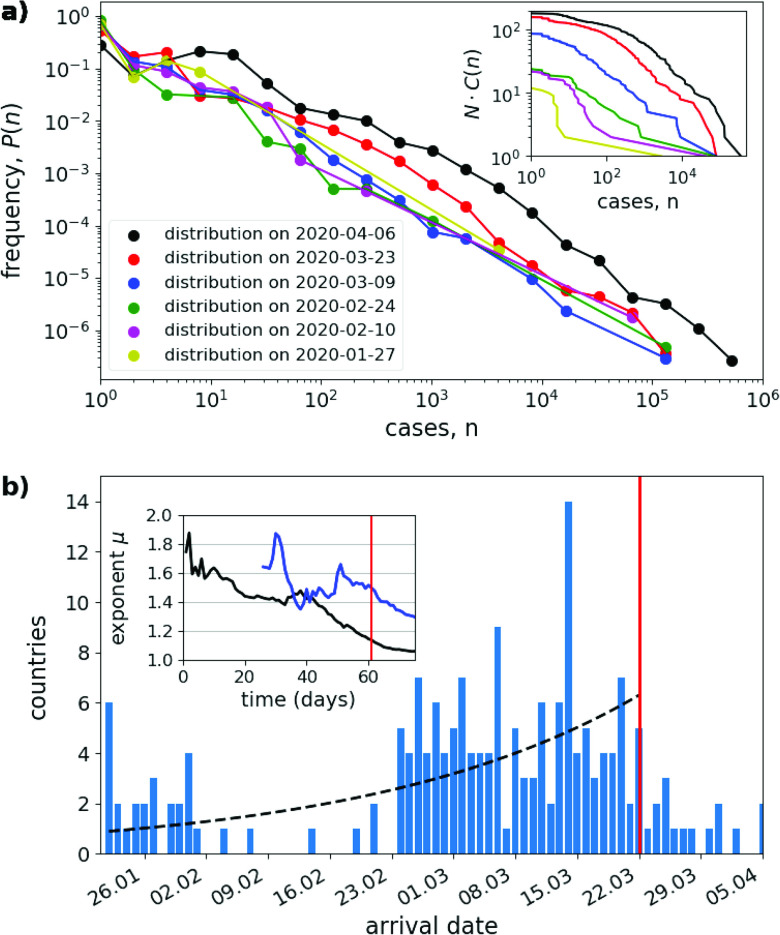
Temporal development of the COVID-19 pandemic. (a) Evolution of the distribution of confirmed cases per country. The same as Fig. 1(a), but for six different time instances separated by 2 weeks (see figure legend) during the pandemic. The inset shows the cumulative number of countries N⋅C(n), where N is the total number of countries with confirmed cases at that date. (b) Distribution of arrival times. The histogram shows the number of countries that were invaded by the virus on a certain day between January 22, 2020 and April 5, 2020 (blue bars). Further shown is an exponentially increasing function, exp⁡(st) (black dashed line) with growth rate $s=0.03\;d^{-1}$, obtained by a least square fit to the histogram during the first 61 days. The inset shows the critical exponents ${\hat{\mu }}_{C}(t)$ (black) and ${\hat{\mu }}_{D}(t)$ (blue, only shown from February 16, the first days with at least five confirmed deaths), estimated by maximizing the log-likelihood function equation (A4), as a function of time. The vertical red line indicates March 22, the date of the distribution shown in Figs. 1(a) and 1(b).

While the present analysis considers the distribution of case numbers at a temporal snapshot, in reality the pandemic is a dynamic process successively invading countries worldwide. In Fig. 2, we investigate the temporal development of the COVID-19 distribution. The figure shows that the country-level distribution of confirmed cases is formed already within a few weeks from the start of the outbreak and remains roughly stationary over the considered time interval of 75 days. A closer inspection (see inset in Fig. 2(b) reveals that the critical exponents in fact are not constant, but in general are decreasing functions of time, indicating that the case distributions tend to broaden over the course of the pandemic.

Figure 2(b) further investigates the spatial spread of COVID-19 across countries worldwide more systematically. The figure plots the number of countries that were invaded by the coronavirus (i.e., having the first confirmed COVID-19 case) at a particular day in the time span from January 22 to April 5, 2020. On January 22, the first entry in the database, six countries (China, Japan, South Korea, Taiwan, Thailand, US) were already invaded by the virus. From this day, within roughly two months, the pandemic spread to nearly every country in the world.

Interestingly, the invasion speed was not constant. Instead, Fig. 2(b) clearly indicates two broad modes in the arrival time distribution. A first group of countries was invaded by the disease in the end of January. In the first three weeks of February nearly no new arrivals were reported. Starting from February 24, a second wave of invasions appeared which lasted until the end of March, after which the number of new arrivals began to fall again, probably reflecting the fact that the pandemic had reached basically all countries of the world. As of April 5, a total 185 countries were invaded by the coronavirus.

There are several possible reasons why the disease arrival is not more evenly distributed. One explanation for the bimodal shape is related to the lockdown of airline transportation in China in the end of January 2020. According to this hypothesis, after the first pandemic bubble in January, the further spread of the pandemic came to a temporary standstill with the onset of travel restrictions, only to resurface in a second wave, starting end of February. Alternatively, it may be that many arrivals of the virus in countries all over the world simply went undetected during the first weeks of February and were detected only later with the increasing awareness and increased testing. This hypothesis is corroborated by the observation that end of February is also the time when the first PCR based tests became available. In general, the strong irregularity in the arrival time distribution points to the high level of stochasticity of the worldwide spreading process.

### C. Mechanistic explanation of the power-law distribution

Figure 2 would suggest that the temporal development of the pandemic is characterized by two complementary processes: the successive invasion of more and more countries and the increasing number of cases within each affected country. Here, we argue that the emergence of the power-law distribution could be related to the concurrent “superposition” of these two processes. Thereby, on a large geographic scale, the pandemic is driven by the spread of the virus in the network of interconnected countries. On a small scale, case numbers are snowballing within each country, once it has been invaded, thereby further increasing the epidemic imbalance due to different arrival times between countries.

In the simplest approximation, at the begin of the pandemic both of these processes developed exponentially in time. A straightforward calculation shows that the combination of the two exponential processes generically yields a truncated power-law distribution in the number of cases in countries: Consider an epidemic outbreak that started (the first case reported in a country) at time t=0. We are interested in the case distribution at time t>0. Let us first assume that at this day, the probability distribution for a country to have been invaded by the virus at some former time τ grows exponential in τ with spreading rate s,

$$P(\tau )∼e^{s\tau },\;0\leq \tau \leq t.$$ (2)

This exponential growth in the geographic distribution of the pandemic would be the expectation if one modeled the spread in a network where nodes are countries (neglecting saturation when the pandemic has reached most countries). Note that the distribution is truncated from two sides because arrivals of the disease can only have occurred after the start of the pandemic, τ≥0, and in the past, τ≤t.

Second, we assume that in each country, the number of confirmed cases has grown exponentially with the time since invasion t−τ with growth rate r (neglecting containment measures and saturation after the epidemic peak),

$$n(t)∼e^{r(t-\tau )}.$$ (3)

Combining these two equations, the probability distribution of confirmed cases P(n) can be calculated as^29^

$$P(n)=P(\tau )\left|\frac{d\tau }{dn}\right|∼\frac{e^{s\tau }}{e^{-r\tau }}∼n^{-(1+s/r)},\;\mathrm{with}\;1\leq n\leq n_{max},$$ (4)

which is a truncated power-law with critical exponent,

$$\mu =1+\frac{s}{r}.$$ (5)

Thus, the critical exponent is simply determined by the ratio of large-scale to small-scale growth rates. In the symmetric case that both growth rates are identical, s=r, we would expect a power-law with μ=2. In the limiting case that the large-scaling spreading process is linear in time, s=0, we obtain a border-line distribution with critical exponent μ=1. Note that from the truncation of τ in the arrival time distribution, Eq. (2), the admissible range of case numbers in the power-law distribution Eq. (4) necessarily is restricted between the lower bound n=1 (the epidemic prevalence in a newly invaded country) and the cut-off value $n_{max}∼e^{rt}$ (the epidemic prevalence at time t in the country with the first confirmed case)—justifying the observation of a truncated power-law in the empirical data as shown in Fig. 1.

Obviously, this simple theory far from accurately describes a real-word pandemic. First of all, the theory is valid only in the initial phase of the pandemic, while both geographical spread and within-country epidemic growth are still exponential. As soon as saturation processes set in, the derivation of the power-law breaks down. Next, as shown in Fig. 2(b), the arrival time distribution during the COVID-19 pandemic is not exponential, as discussed above. In gross oversimplification, we may nevertheless fit an exponential function $P(t)∼e^{st}$ through the data, yielding an “average” spreading rate of $s=0.03\;d^{-1}$ [black dashed line in Fig. 2(b)]. Finally, epidemic growth rates during the COVID-19 pandemic have not been identical in all countries (even in the initial stages). They have also not remained constant in time, but in most countries have fallen in the course of the epidemic. Furthermore, most countries were invaded multiple times, leading to different epidemic foci within countries. Neglecting all these observations, for the sake of argument, let us assume an average doubling time of case numbers of $T_{1/2}=3.5\;d$ in all countries, yielding an exponential growth rate of $r=\log(2)/T_{1/2}=0.2\;d^{-1}$ and a maximal case number of $n_{max}=e^{0.2*60}=1.6\times 10^{5}$ after 60 days. Then, according to our simple theory equation (5), we would expect a critical exponent of μ=1+0.03/0.2≈1.15, in rather good agreement to the fitted exponents in Fig. 1.

### D. Results from a meta-population model

To test the theory of Sec. II C, we developed a dual-scale meta-population model (see Appendix B). The first level describes the large-scale stochastic spread of the virus in a network of N interconnected countries. The second level describes the small-scale increase in case numbers within a country; it is started in each country from the time point of invasion by the virus and follows a simple deterministic SIR-dynamics. The motivation for this model design was not to predict the worldwide spread of COVID-19, but rather to quantitatively test the emergence of heterogeneous case distributions in a conceptual model framework that incorporates the ideas from Sec. II C.

**FIG. 3.**
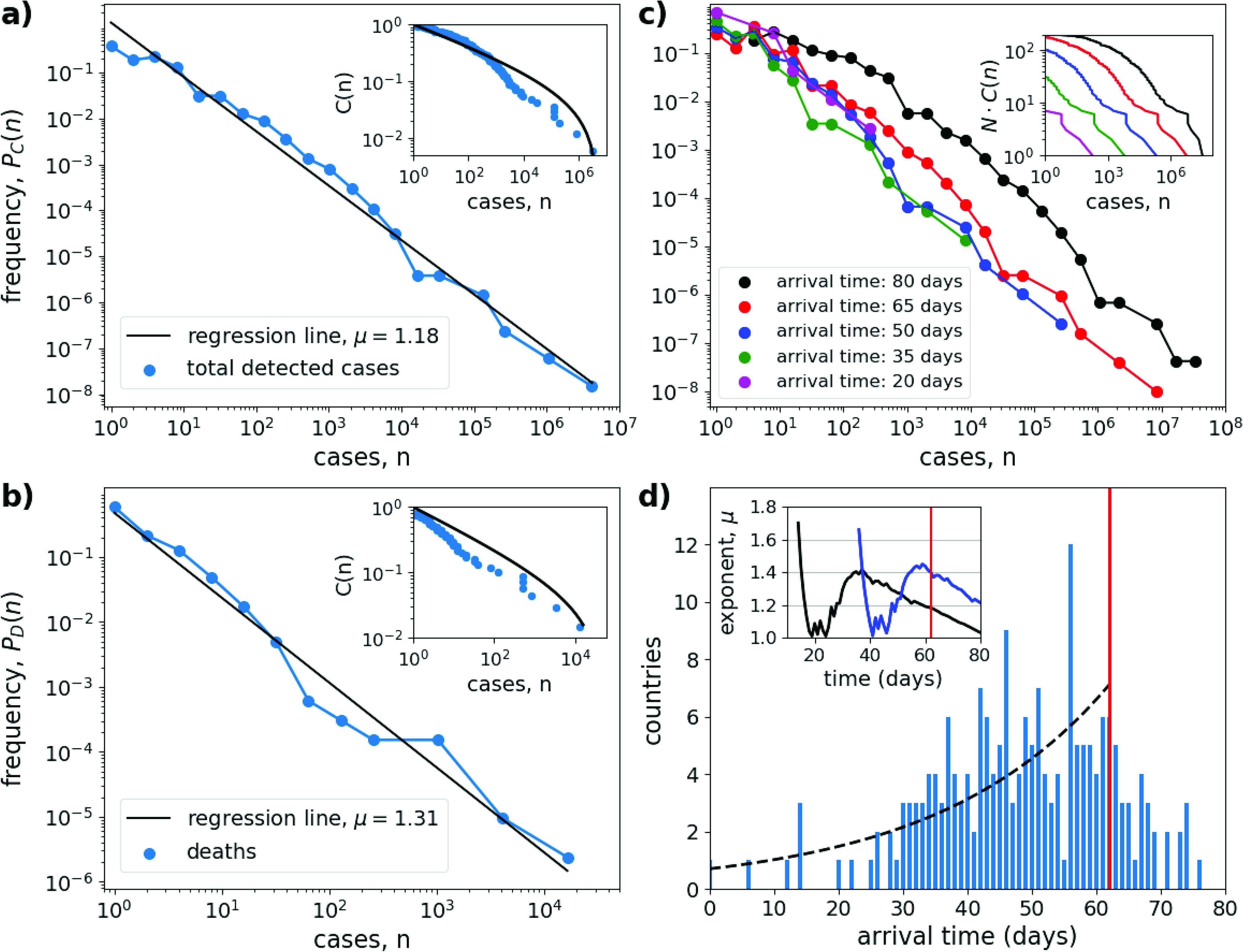
Power-law scaling in the meta-population model. (a) and (b) Same as Fig. 1, but for model simulations after transient of 62 days. Shown is the estimated probability P(n) (blue lines and circles) with straight-line fits (black lines) for the simulated cases (a) and deaths (b). Insets show the cumulative fraction C(n) of countries (blue circles) and the cumulative distribution function (black lines) of a truncated power-law distribution with cut-off values (a) $n_{max}=5\times 10^{6}$ and (b) $n_{max}=5\times 10^{4}$. (c) and (d) Same as Fig. 2, showing the spatial spread in the meta-population model. (c) Evolution of the distribution of cases as in (a), but for five different time instances separated by 15 days (see figure legend). (d) Histogram of arrival times, showing the number of countries that were invaded on a certain day. Simulation time: 80 days. The black dashed line shows an exponentially increasing function, exp⁡(st) with spreading rate $s=0.037\;d^{-1}$, obtained by a least square fit to the data during the first 62 days. The inset shows the time dependence of the critical exponents ${\hat{\mu }}_{C}(t)$ (black) and ${\hat{\mu }}_{D}(t)$ (blue) for the distribution of the number of cases and deaths, estimated by maximizing the log-likelihood function equation (A4) for all days where at least five cases were reported. The red vertical line indicates day 62, the time of the distribution in (a) and (b). See Appendix B for model description and parameter values.

Figure 3 shows a typical model outcome. The large-scale spreading process is captured in the arrival time distribution, which exhibits a unimodal dependency on time [Fig. 3(d)]. Correspondingly, the number of invaded countries grows stochastically and roughly follows a sigmoidal shape. In accord to our theory, Eq. (2), this arrival time distribution starts to grow exponentially in the build-up phase of the pandemic. The highest invasion rates occur after about 50 days, while after a simulation time of 80 days, 196 out of the N=200 countries are already invaded by the virus.

Combining the large-scale and small-scale model components allows us to simulate the epidemic prevalence in each country as a function of time. Figures 3(a) and 3(b) show the resulting distribution of cases and deaths after a simulation time of 62 days [vertical red line in Fig. 3(d)]. Again, the distributions are well characterized by a truncated power-law. Comparison with Fig. 1 shows that the model is able to describe the characteristics of the empirical distribution of COVID-19 cases rather well. The log-likelihood estimation of the critical exponents yields values of ${\hat{\mu }}_{C}=1.18\pm 0.01$ and ${\hat{\mu }}_{D}=1.40\pm 0.06$. These exponents can be compared to our theory equation (5). From Fig. 3(d), we estimate a spatial spreading rate of $s=0.037\;d^{-1}$ in the build-up phase of the pandemic. The initial growth rate of infected in the SIR-model equals $r=0.23\;d^{-1}$ (see Appendix B). Thus, according to Eq. (5), we would expect a critical exponent of μ=1+0.037/0.23=0.16, in good agreement to the estimated value from the numerical simulation.

We want to note that the nearly ideal power-law scaling in the case distribution holds only in the initial phase of the pandemic and is lost when the spatial spreading starts to saturate. This can be seen in the simulated case distribution P(n) for different time instances [Fig. 3(c)]. While P(n) remains roughly stationary for the first 50–60 days of the simulation, a first plateau begins to emerge at the left end of the distribution for larger times. This plateau reflects the fact that when the number of newly invaded countries is reduced, these countries with just a few cases are missing in the left end of the case distribution [reminiscent to the behavior exhibited in the empirical case distribution, Fig. 2(a)]. Additionally, the estimated critical exponents are decaying in time [inset of Fig. 3(d)], similar to that of the empirical data [inset of Fig. 2(b)]. Thus, the first sign that the outbreak has reached most countries in the network is the reduction in the scaling range and a simultaneous broadening of the case distribution. Eventually, in the limit of large time, when the epidemic has come to an end in every country, scaling is lost and the distribution of cases must converge toward a delta function $P(n)=\delta (n-fN_{pop})$, with f the fraction of susceptible out of a population of $N_{pop}$ individuals in a country that will be infected (or it would approach the country size distribution in a meta-population with heterogeneously distributed country sizes). Interestingly, in our numerical simulations, we still obtained power-law distribution when the contact rate β was set to a large value, so that the dynamics within a country rapidly reach a stationary state. In this case, with increasing β (and thus increasing initial epidemic growth rates r), the critical exponents tended to μ→1.

## III. DISCUSSION

It is well known from the literature^11,29^ that caution is in order when trying to identify power-law distributions in real data and, in particular, that a straight line in a double-logarithmic plot does not suffice to prove the existence of a power-law distribution. For this reason, the aim of this study is not to prove that the COVID-19 case distribution is a perfect power-law, an undertaking that would require sophisticated statistical analysis and a much larger sample size.^11^ We also do not intend to rule out other likely candidate distributions (e.g., log-normal or stretched exponential distributions). Instead, our claim is merely to demonstrate that the empirical data are highly consistent with the hypothesis that the number of reported cases are taken from a truncated power-law distribution of the form equation (1).

Nevertheless, the scaling relations in the distributions shown in Fig. 1 are remarkably constant over the whole range of case numbers, stretching several orders of magnitude with no obvious signs of saturation for either the range of small or large case numbers. One might argue that the bend in the cumulative distribution function is a sign that the growth in some countries (e.g., China, Korea) had already become sub-exponential. However, this is contradicted by the observation that a similar bend is also exhibited by the cumulative distribution function obtained from the meta-population model (Fig. 3). Thus, the most likely explanation is that the case distribution follows a truncated power-law (see also Fig. 5), suggesting the hypothesis that the spatial distribution of COVID-19 cases is a fractal.^9^ This is further corroborated by our simple theory, which provides a mechanistic explanation for why we would expect a truncated power-law in the first place.

Our finding of power-law distributions in the number of reported cases has important consequences for epidemiology. Most notably, the small values of the estimated critical power-law exponents are related to the strong inequality of case numbers that was frequently observed all over the world in the initial phase of the COVID-19 outbreak. Following a power-law distribution means that this pattern prevails even as numbers grew and the scale of infection expanded globally. In particular, during the course of the pandemic, most cases were reported to have occurred in a few countries, sometimes even a single country—the so-called epicenters of the pandemic. The distribution of cases within countries followed a similar pattern. Often COVID-19 was peaking in a few localized foci (local regions or cities), while other parts of the country at the same time had experienced only a moderate number of cases. Our theory provides a mechanistic explanation why this might have been the case.

**FIG. 4.**
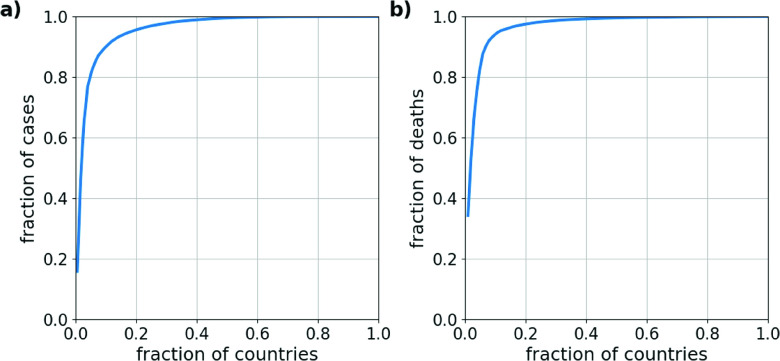
Lorenz curves, depicting the inequality in the distribution of confirmed COVID-19 cases. The plots show the fraction of the number of confirmed cases (a) and of the number of confirmed deaths (b) as a function of the fraction of most affected countries on March 22, 2020 (compared to Fig. 1). This inequality corresponds to a Gini-coefficient of G=0.92 for the distribution of confirmed cases and of G=0.94 for the number of confirmed deaths.

A graphical representation for the inequality of a distribution is given by the Lorenz curve,^29^ which in the case of the COVID-19 case distribution is a plot of the fraction of the total number of confirmed cases in dependence of the fraction of the most affected countries. This is shown in Fig. 4 for the number of confirmed COVID-19 cases and confirmed deaths on March 22, 2020. The Lorenz curve shows that on this day, 95.7% of confirmed cases and 97.6% of the confirmed deaths had been reported in the 20% most affected countries (while the top 5% most affected countries had accumulated 82.3% of all confirmed cases and 84.4% of all confirmed deaths). With 81 435 out of 336 953 confirmed cases on that day, China alone had accumulated a fraction of 24% of all cases. The two most affected countries, China and Italy, together had accumulated a fraction of 41% of the worldwide reported cases.

**FIG. 5.**
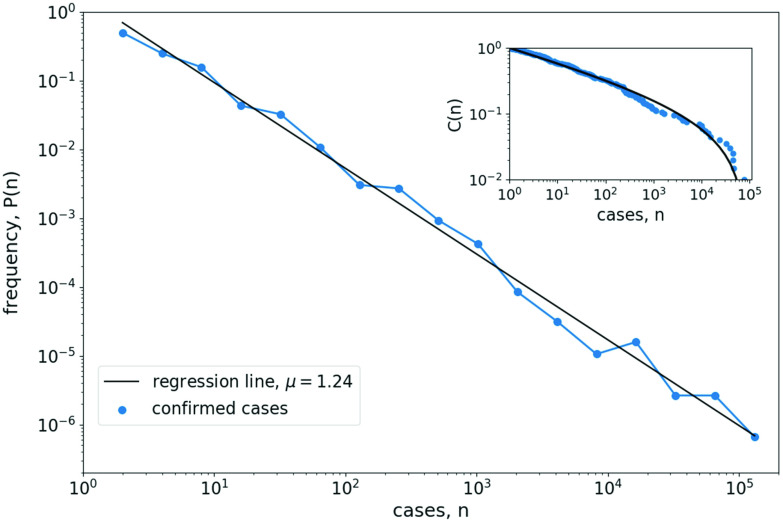
Robustness of the algorithm for estimating parameters of a truncated power-law. The same as Fig. 1 but for 200 random numbers $n_{i}$ that were generated from a truncated power-law distribution with μ=1.2 and cut-off value $n_{max}=1\times 10^{5}$. The estimated distribution roughly follows a straight line on the double-logarithmic plot with equally spaced bins. Note that even though only 200 random numbers were drawn, the estimated probabilities vary over many orders of magnitude (which is numerically possible since in order to compute the probability distribution, the histogram counts are divided by the variable bin sizes). Log-likelihood estimation of critical exponent (Appendix A) yields $\hat{\mu }=1.21\pm 0.01$ in good agreement with the actually used exponent. In contrast, the estimator for an unbound power-law, Eq. (A6), yields $\hat{\mu }=1.28\pm 0.01$, strongly overestimating the true exponent. The estimation by a regression line, yielding μ=1.24, also is slightly too large. The inset shows that the cumulative fraction is well described by the cumulative distribution function C(n) of a truncated power-law with critical exponent $\hat{\mu }$ and $n_{max}=8.4\times 10^{4}$, the maximal $n_{i}$ value of the sample.

This inequality can also be measured by the Gini-coefficient G,^17^ which ranges between G=0 for perfect equality, i.e., all countries having the same number of cases, and G=1, corresponding to maximal inequality, where all cases appear in a single country. For the distribution of confirmed COVID-19 cases on March 22 [Figs. 1(a) and 1(b)], we obtain a Gini-coefficient of G=0.92 and for the number of confirmed death of G=0.94. These large values are a direct consequence of the small critical exponents of the estimated power-law distributions. In fact, for an unbounded power-law distribution with μ<2, one would theoretically expect a Gini-coefficient of G=1.^29^

The emergence of power-law distributions with a small critical exponent and the associated inequality of the distribution, with Gini coefficients close to one is also observed in the developed meta-population model. Consequently, also in the model case numbers are mostly concentrated in a few countries. In the simulations, these epicenters of the pandemic, i.e., the countries with most cases, are always the countries in which the diseases originated or which were first invaded by the virus. In other words, the prevalence rank order among countries remains unchanged during the course of the pandemic. This is akin to the “rich-get-richer process” or “first-mover-advantage,”^30,38^ a well-studied process to generate power-law distributions. In the real COVID-19 pandemic, this was not the case. During the beginning of the pandemic, most cases were observed in China, later the “leading role” changed next to Italy and finally to the USA. This reflects different mitigation strategies and circumstances in different countries, a factor that is not considered in the simple model. Nevertheless, despite these changes in the rank order, the distribution of cases in the empirical data was always closely represented by a power-law.

We would like to remark that the available database only provides information on the reported COVID-19 cases in each country. In all likelihood, the real number of cases will be much larger. Not much is known about the reporting rates, but first estimates indicate that a substantial fraction (possible 86%) of infections might go undetected.^24^ Reporting rates probably vary strongly between countries and may change in time with the awareness of national health institutions and available testing capabilities. Further uncertainties arise because the criteria by which a person is classified as active case (and even more so for being classified as recovered) vary between countries and not uncommonly have been modified during the course of the pandemic within a country.

Remarkably, we obtained power-law distributions in the absolute number of cases in each country. At first guess, one might have expected such scaling only after case numbers have been normalized by population sizes. Our preliminary investigations show that such normalized case numbers become even more unequally distributed, with even smaller estimated values of the critical exponent, and the distributed values do not line up any more so well on a straight line on a double-logarithmic plot. Thus, “folding” the distribution of population sizes over the COVID-19 case distribution does not flatten, but rather tends to further increase, the inequality of the resulting distribution. This indicates that absolute (non-normalized) case numbers may be the natural variables to describe the patterns of the pandemic in its initial stage. In all likelihood, the role of country sizes and population numbers will become increasingly important with the further spread of the pandemic.

We have shown that a simple conceptual model yields an accurate description of the COVID-19 prevalence distribution in the initial phase of the pandemic. This is remarkable because many important epidemiological aspects of the spreading process are not captured by the model. Most notably, the model takes into account neither variability in country sizes, population numbers, testing rates, heterogeneity of intra- and inter-country connectivity, nor the corresponding changes due to social distancing, lock-down measures, closing of airline connections and shut-down of borders.

These simplifications leave much room for future investigations and model improvements. Obvious model improvements would be to consider a meta-population with heterogeneously distributed country sizes or to make the initial number of infected individuals a random number, as would be a better description of what happened in many countries.

One basic assumption of the developed model is the separation of the pandemic into two spatial scales, the large-spatial spread over a rather small number (N<200) of interconnected countries and the small-scale growth within a population of much larger size ($N=5\times 10^{7}$). This separation obviously is somewhat arbitrary. For the virus, countries are, of course, quasi-arbitrary entities. Therefore, it would be important to check whether both the data analysis (Fig. 1) and the mathematical model are robust to arbitrarily subdividing or lumping countries. The very similar scaling observed among US counties [Figs. 1(c) and (d)] lends credence to the model’s generality. Similarly, one can readily ascertain that the model result is not an artifact of artificial lumping. Suppose a virus that is spreading in an all-to-all, or randomly coupled, network of a number of $N\cdot N_{pop}$ individuals. If we would artificially subdivide individuals into a small number N of classes (or countries), at the time point when the disease has spread to all countries, within each country, we would still have only a few cases (of the order of $N\ll N_{pop}$). Thus, the assumed simultaneous spread on both spatial scales requires a real physical separation in the network structure. It would be an interesting perspective for future research to study the spread in multi-scale hierarchies or in more realistic models of interconnected societies.

One important model application would be the simulation of interim COVID-19 lockdown or containment measures, as were introduced in many countries in the world in March and April 2020. Such measures might inhibit the increase of case numbers within local regions (the small-scale part of our theory) but they would not necessarily suppress also the large-scale diffusion of infections across regions. Thus, under the guise of suppressed case numbers during the mitigation period, there could be a dangerous “invisible” homogenization in the spatial distribution of the virus. This would have tremendous implications in a scenario where the measures are suddenly lifted in many places. In this case, our theory would predict the emergence of a very different case number distribution than shown in Fig. 1. Instead of the previous power-law distribution resurfacing, the most likely situation would be the synchronous initiation of increasing in case numbers everywhere. Thus, situations as they appeared only in the epicenters during the beginning phase of the pandemic could be the rule in most parts where mitigation measures are lifted. In this sense, the long tail of the case distribution, characterized by the many regions with only mild epidemic prevalence, that was observed in the initial phase of the pandemic, could create a false sense of security.

Finally, we would like to remark that the model’s strong simplicity is at the same time a strength: being rather generic, it should be applicable to very different systems, to describe the spread of commodities as a process with two spatial scales. The fact that the distribution of COVID-19 resembles a model where only the initial infection “counts” reflects the intrinsic difficulty in containing epidemics at global and local scales when unilateral measures (e.g., travel bans and lockdowns) are impractical or non-enforceable, i.e., where other countries or regions will step up and continue the spread. Thus, assessing how a well simple dual-scale model predicts the early spread of epidemics, despite the huge contrasts between countries, could help identify critical temporal and spatial scales of response in which to mitigate future epidemic threats.

## Figures and Tables

**FIG. 6.**
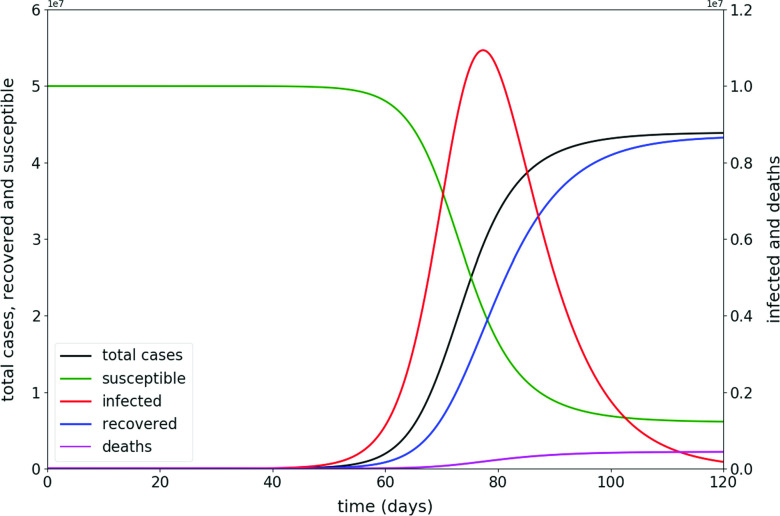
Simulation of the SIR-model (B1) within a country. The plot shows the numerically obtained values of the total number of cases C (black), the number of susceptible S (green), and the number of recovered R (blue) on the left axis, as well as the number of infected I (red) and deaths D (magenta) and the right axis as a function of time. For the used initial values of $S(0)=5\times 10^{7}$, I(0)=1, and R(0)=D(0)=0, the epidemic peak is reached after 77 days. See methods for parameter values.

**FIG. 7.**
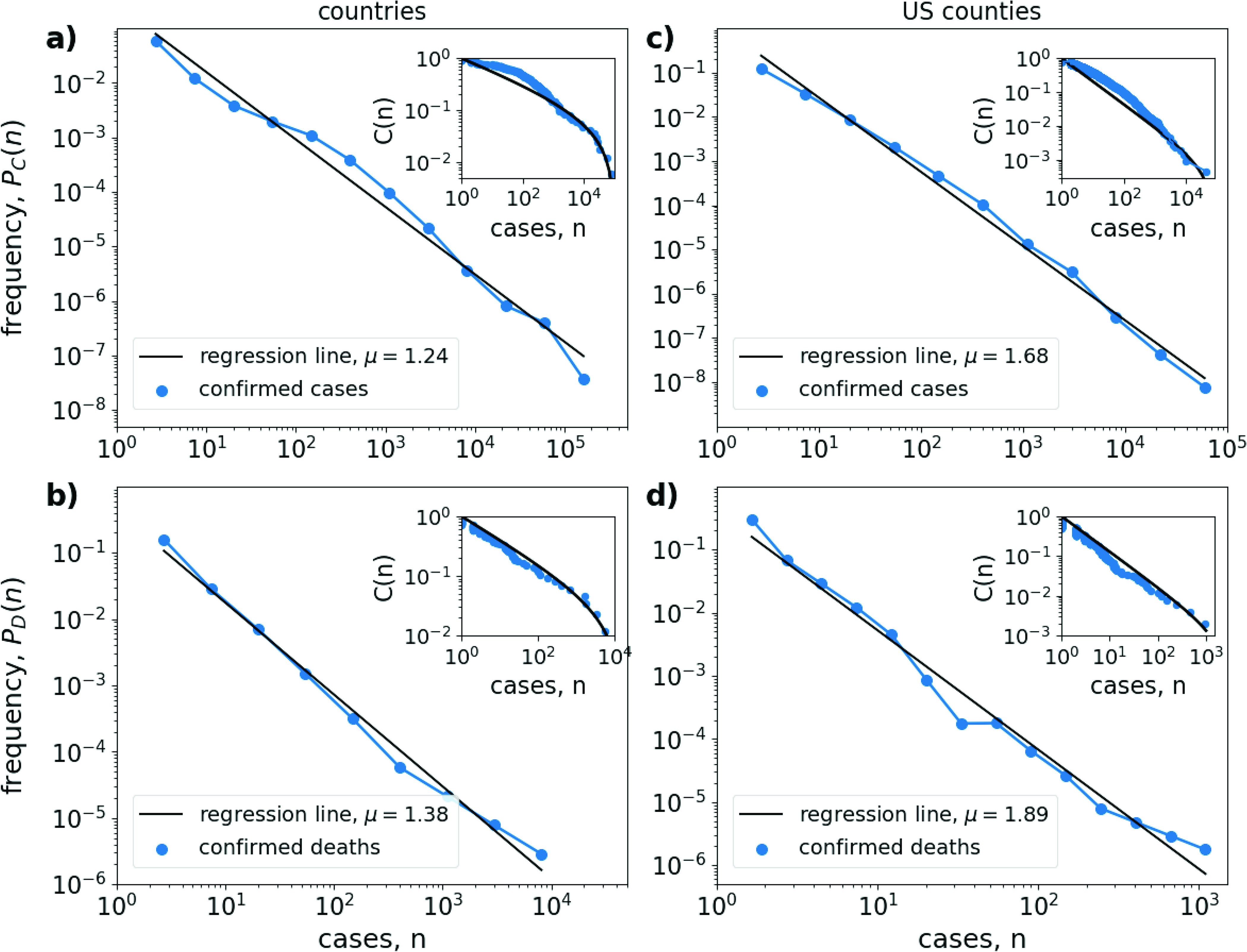
Alternative calculation of the distribution of COVID-19 case numbers in countries worldwide and counties in the US. Same as Fig. 1, but for the alternative histogram algorithm, using a histogram of log-transformed case numbers (see Appendix A). Left column: Estimated probability $P_{x}(n)$ (blue lines and circles) for a country to have a certain number n of (a) confirmed cases (x=C) and (b) confirmed deaths (x=D) on March 22, 2020. Right column: The same for the 2160 US counties that have been invaded by the coronavirus on March 31, 2020, for (c) confirmed cases and (d) confirmed deaths. Insets: Cumulative fraction $C(n)=\sum_{m=n+1}^{N}P(m)$ of countries, or counties, with case number m>n.

## Data Availability

Code for data analysis and numerical simulations was written in Julia, Ref. 6, available in https://github.com/berndblasius/Covid19. The differential equations were solved with the package DifferentialEquations.jl, available in https://github.com/berndblasius/Covid19, Ref. 35 and the maximization of the log-likelihood function with package Optim.jl, available in https://github.com/berndblasius/Covid19, Ref. 28. The used data are available in https://github.com/CSSEGISandData.

## APPENDIX A: ESTIMATING PARAMETERS OF A TRUNCATED POWER-LAW DISTRIBUTION

Assume a truncated power-law (or Pareto) distribution of the random variable n

$$P(n)=Cn^{-\mu },\;1\leq n\leq n_{max},$$ (A1)

with upper bound $n_{max}$. Normalization ∫P(n)dn=1 yields $C=(1-\mu )/(n_{max}^{\left(1-\mu \right)}-1)$. The cumulative distribution function reads

$$C(n)=\int_{n}^{n_{max}}P(n^{\prime })\;dn^{\prime }=\frac{n^{\left(1-\mu \right)}-n_{max}^{\left(1-\mu \right)}}{1-n_{max}^{\left(1-\mu \right)}}.$$ (A2)

In the limit $n_{max}\to \infty$ (a power-law distribution without upper bound), the cumulative distribution function also follows a power-law $C(n)∼n^{1-\mu }$.

A synthetic sample of the distribution (A1) can be obtained by the formula

$$n_{i}={\left[1-u_{i}(1-n_{max}^{\left(1-\mu \right)})\right]}^{\frac{1}{1-\mu }},$$ (A3)

where $u_{i}$ are random numbers taken from a uniform distribution in the range [0,1].

The inverse problem is to estimate the parameters of the distribution given a random sample $n{(}_{1},n_{2},\ldots ,n_{N})$ of N data points. The log-likelihood for the distribution Eq. (A1) can be defined as^1,13,45^

$$L(\mu )=\sum_{i=1}^{N}\ln P(n_{i})=N\ln\left(\frac{1-\mu }{n_{max}^{\left(1-\mu \right)}-1}\right)-\mu \sum_{i=1}^{N}\ln n_{i}.$$ (A4)

Then, an estimator for the cut-off value is obtained by ${\hat{n}}_{max}=\max(n_{i})$^1^ and an estimator $\hat{\mu }$ for the critical exponent is obtained by maximizing L(μ), yielding a standard error^13^

$$\sigma =\frac{1}{\sqrt{N}}{\left[\frac{1}{{\left(\hat{\mu }-1\right)}^{2}}-\frac{{\hat{n}}_{max}^{\left(\hat{\mu }-1\right)}\ln^{2}{\hat{n}}_{max}}{{\left(1-{\hat{n}}_{max}^{\left(\hat{\mu }-1\right)}\right)}^{2}}\right]}^{-\frac{1}{2}}.$$ (A5)

In these expressions, the “hat” means that we refer to an estimated value. The maximization of L(μ) must be computed numerically (here, we use Brent’s method from the Julia package Optim.jl^28^).

In the limit of an unbounded power-law distribution $n_{max}\to \infty$, the maximization can be calculated analytically, yielding^29^

$$\hat{\mu }=1+N{\left(\sum_{i}\ln n_{i}\right)}^{-1}.$$ (A6)

To estimate the distribution P(n) of case numbers that vary over many orders of magnitude, we used a histogram with logarithmic binning. That is, we placed a discrete number of bins, k, at positions of integer powers of two $n_{k}=2^{k}$ (i.e., 1, 2, 4, 8, 16, etc.) and for each bin counted the number $H_{k}$ of countries (or US counties) that under the day of investigation reported a number n of cases that was falling into this bin ($n_{k}\leq n<n_{k+1}$). To obtain the probability distribution, the resulting histogram counts were divided by the varying bin sizes $P(n_{k})=cH_{k}/(n_{k+1}-n_{k})$ and the normalization constant c fixed so that $\sum_{k}P(n_{k})=1$. For visualization, we plotted the distribution on double-logarithmic axes, excluding bins without entries.

To confirm the robustness of the histogram estimation, we also used an alternative algorithm, where we first computed the histogram of log-transformed case numbers ν=log⁡(n) using equally spaced bins, which, after normalization, yielded the distribution $\tilde{P}(\nu )$. Next, we used the back-transform $P(n)=\tilde{P}(\nu )/n$ to obtain the probability distribution P(n) of non-logarithmic case numbers. This procedure also yields a distribution with bins that are equally spaced on a logarithmic scale and the resulting distributions, shown in Fig. 7 in Appendix C, are very similar to that from the logarithmic binning method described above. We have checked that the resulting distribution is largely independent to the choice and number of used histogram bins and other numerical parameters.

We also computed the cumulative fraction $C(n)=\sum_{m=n+1}^{N}P(m)$ of countries with case number m>n. This was obtained by taking a rank-plot of case numbers and inverting axes, i.e., sorting the array of case numbers in descending order and plotting for each country the rank as a function of the sorted case number on double-logarithmic axes.^29^

## APPENDIX B: THE METAPOPULATION MODEL

To describe the spatiotemporal evolution of epidemic prevalence during the course of a pandemic, we developed a conceptual dual-scale meta-population model. The large-scale model component allows us to simulate the spread of the virus in a network of N interconnected countries (with average network degree k). The state of a country is given as a Boolean value, being either invaded by the virus or non-invaded. The model starts with a single invaded country. The geographic spread runs in discrete time, each step corresponding to one day of the time-continuous small-scale model. In each time step, a non-invaded country becomes infected by neighboring invaded countries in the network with the transmission probability p. As soon as a country has been invaded by the virus in this process, the small-scale model for this country is initiated.

This large-scale model corresponds to the well-known SI-epidemic spread on a network.^31^ The number of invaded countries grows stochastically and roughly follows a sigmoidal shape. Neglecting saturation effects (that is, in the initial phase of the pandemic) and assuming a homogeneous degree distribution, the expected number of invaded countries X(t) grows exponentially in time, $X(t)=X_{0}\exp(st)$, with $X_{0}=1$ and exponent s=kp. Then, the rate of newly invaded countries is given by $\dot{X}=s\;\exp(st)$ and thus also the probability distribution P(τ) for a country to have been invaded at some former time τ≤t, Eq. (2), grows exponentially, P(τ)=cexp⁡(st). Here, the normalization factor is given by $c=\frac{s}{N}\exp(st)/(\exp(st)-1)$, which is determined by the condition that the time integral $\int_{0}^{t}P(\tau )\;d\tau =X(t)/N$.

In our simulation, parameter values were taken as follows: number of countries N=200, degree k=199 (fully connected network), and invasion probability $p=6\times 10^{-4}$.

The small-scale model is time-continuous and deterministically describes the epidemic dynamics within a country. The model determines the time course of susceptible S, infected I, recovered R, and dead D from a standard SIR-model,^22,23^

$$\dot{S}=-\beta \frac{S}{N_{pop}}I,\;\dot{I}=\beta \frac{S}{N_{pop}}I-\gamma I,\;\dot{R}=(1-m)\gamma I,\;\dot{D}=m\gamma I.$$ (B1)

Here, $N_{pop}$ is the constant population size in the country, β is the contact rate, 1/γ the infectious period, and m the case fatality rate. The total number of cases is determined as C=I+R+D. In the small-scale model, countries are simulated independently from each other and are only coupled by the unique invasion event for each country, which starts the epidemic growth in that country with initial values $S(0)=5\times 10^{7}$, I(0)=1 and R(0)=D(0)=0. All infection state variables in a country are zero before invasion by the virus, I=R=D=0. The resulting well-known SIR-dynamics in a single country is shown in Fig. 6. With the chosen parameterization, it takes roughly 80 days until the epidemic peak is reached. After this time, the assumption of an exponential increase, Eq. (3), breaks down. Parameter values were taken as follows: country population size $N_{pop}=5\times 10^{7}$, case fatality rate m=0.01, infectious period 1/γ=6d, and contact rate $\beta =0.4d^{-1}$. This yields a growth rate $r=\beta -\gamma =0.23d^{-1}$, corresponding to a doubling time of $T_{1,2}=\log(2)/r=3d$ and a basic reproduction number $R_{0}=\beta /\gamma =2.4$.

## APPENDIX C: SUPPLEMENTARY FIGURE

Alternative calculation of the distribution of COVID-19 case numbers in countries worldwide and counties in the US.
